# Network Pharmacology and Experimental Validation Reveal Therapeutic Potential of Propolis in UV-Induced Allergic Dermatitis

**DOI:** 10.3390/foods14060996

**Published:** 2025-03-14

**Authors:** Liyuan Cheng, Jie Wang, Yicong Wang, Jingjing Li, Wenchao Yang

**Affiliations:** 1 College of Bee Science and Biomedicine, Fujian Agriculture and Forestry University, Fuzhou 350002, China; clyuan0819@163.com (L.C.); wangjie01092023@163.com (J.W.); 18265526098@163.com (Y.W.); lijingjing000407@163.com (J.L.); 2 College of Food Science, Fujian Agriculture and Forestry University, Fuzhou 350002, China

**Keywords:** propolis, UV-induced allergic dermatitis, treatment strategy, network pharmacology, in vitro

## Abstract

Propolis demonstrates diverse pharmacological properties encompassing antimicrobial, anti-inflammatory, antioxidant, immunomodulatory, and wound-healing activities. This study investigated the therapeutic mechanism of propolis against ultraviolet (UV)-induced allergic dermatitis through an integrated approach combining network pharmacology with in vitro experimental validation. The targets of propolis components were conducted through the PubChem, the EMBL-EBI, and SEA Search Server databases, and the disease-associated targets for atopic dermatitis and related allergic conditions were extracted from GeneCards. The overlapping targets between propolis components and UV-induced dermatitis were screened. The Gene Ontology (GO) Enrichment analysis and Kyoto Encyclopedia of Genes and Genomes (KEGG) pathway enrichment analysis were performed. The key targets were further validated through ELISA experiments using HSF cells. The results show that there were 28 overlapping targets between propolis and UV-induced allergic dermatitis. The GO enrichment results show that there were 1246 terms of biological functions, 52 terms of cellular components, and 98 terms of molecular functions. KEGG pathway enrichment obtained 110 signaling pathways. The protein–protein interaction (PPI) network showed that TNF, NFKB1, MMP-9, and IL-2 were hub proteins. The ELISA experiment confirmed that propolis reduced the levels of MMP-9 and IL-2 in UBV-induced allergic dermatitis of HSF cells in a dose-dependent manner. These findings provide mechanistic evidence supporting propolis as a promising functional food, dietary supplements, or medicinal agent for UV-induced allergic skin disorders.

## 1. Introduction

Ultraviolet (UV)-induced allergic dermatitis, commonly known as photoallergy, is an inflammatory skin disease caused by an abnormal reaction to sunlight [1]. The disease may be caused by various factors, including genetic susceptibility, immune system dysfunction, or the interaction of sunlight with certain chemicals, drugs, or plant-derived substances (phototoxicity or photoallergy) [2,3]. The pathogenesis of UV allergic dermatitis involves a complex interaction between UV radiation and the biological components of the skin [4,5,6], which cause acute or chronic skin damage, including sunburn, inflammation, photo-immunosuppression, photoaging, and even skin cancers [2,6]. Photodermatitis can seriously affect the quality of life, especially for people who are sensitive to sunlight or live in areas with high UV radiation [7]. UV allergic dermatitis is often associated with underlying diseases such as lupus erythematosus, polymorphic light eruption, or certain genetic diseases such as xeroderma pigmentosum [8]. In addition, environmental and lifestyle factors, such as using photosensitizers (e.g., psoralens, nonsteroidal anti-inflammatory drugs, or fragrances), further increase this risk [9,10,11]. Current treatment strategies for photodermatitis focus on prevention and symptom management [12], including broad-spectrum sunscreens, protective clothing, antihistamines, corticosteroids, and, in severe cases, immunosuppressants [13,14]. Recent advances in photobiology have also stimulated the search for novel therapies to mitigate UV-induced skin damage, such as photoprotection enhancers and antioxidant formulations [15]. Propolis has the potential to be one of the new treatment strategies for UV-induced allergic dermatitis.

Propolis is a solid colloidal substance made by western bees collecting resins from various parts of plants and mixing them with their secretions. Propolis contains about 40–45% resins, 25–30% fatty acids, 10% essential oils, 5% pollens, and 5% minerals with other compounds of organic and inorganic nature [16,17], which contribute to multiple biological properties and functions, including antibacterial, antioxidant, antiviral, antitumor, and anti-inflammatory activities [18,19,20,21,22]. Artepillin C, flavonoids, phenolic acids, and terpenes are the main active substances in propolis [22]. The flavonoid compounds in propolis mainly include quercetin, galangin, kaempferol, catechins, and chrysin, which have multiple biological activities such as anti-inflammatory, antibacterial, and antioxidant activities [17,23,24]. Propolis is also rich in phenolic acid compounds, including gallic acid, coumaric acid, caffeic acid, and cinnamic acid, which have antioxidant, lipid-lowering, and wound-healing-improving effects [25,26,27]. A large number of studies have confirmed that propolis and its active ingredients have significant anti-inflammatory effects [20,26,27,28]. Caffeic acid phenethyl ester (CAPE) can inhibit the activation of the NE-κB signaling pathway [29]. Caffeic acid, a phenolic compound that is frequently present in propolis, diminishes nitric oxide (NO) and prostaglandin E2 (PGE2) production in LPS-stimulated RAW264.7 cells [30]. Brazilian red propolis decreases IL1α, IL1β, IL4, IL6, IL12p40, Il12p70, IL13, MCP1, and GM-CSF production [30]. Topical Sydney propolis can prevent inflammation caused by ultraviolet allergy [31]. Ethanolic extract of propolis with CAPE and CAPE alone significantly inhibit carrageenin oedema, carrageenin pleurisy, and adjuvant arthritis [32]. The expression of proinflammatory interleukins (ILs), such as IL-8, IL-12, IL-1β, tumor necrosis factor alpha (TNF-α), cyclooxygenase-2, and inducible nitric oxide synthase are significantly decreased in pretreatment with Korean propolis via the suppression of mitogen-activated protein kinases and nuclear factor κB (NF-κB) pathway [33]. Ethanol extract of propolis and its constituent CAPE inhibit TLR4 signal pathway in breast cancer cells within an inflammatory microenvironment [34]. Our previous report also found that Brazilian green propolis ethanol extracts could reduce LPS-induced inflammatory reactions, improve cell survival, and protect mouse aortic endothelial cells by regulating the proteins and gene expression of ICAM-1, VCAM-1, and MCP-1 [35]. However, the impact and specific molecular mechanism of propolis in the treatment of UV allergic dermatitis have not yet been fully elucidated.

To fill the gap between propolis and UV allergic dermatitis, network pharmacology [36] was employed to deeply explore the potential mechanism of action of propolis in the treatment of UV allergic dermatitis, systematically reveal the complex relationship between propolis components and UV allergic dermatitis-related targets, and provide scientific basis for propolis as a promising functional food or dietary supplement for UV-induced allergic skin disorders.

## 2. Materials and Methods

### 2.1. Retrieve the Targets of UV Allergic Dermatitis and the Main Components of Propolis

First, the targets of the main components of propolis in EMBL-EBI (https://www.ebi.ac.uk/chembl/, accessed on 24 December 2024) and SEA Search Server (https://sea.bkslab.org/, accessed on 24 December 2024) were counted. Then, the “Ultraviolet allergic dermatitis” keyword-related targets were searched for in the “Genecard” database (https://www.genecards.org/, accessed on 24 December 2024), and effective targets with a score greater than 10 were screened. The target-related information was proofread in the UniProt database (https://www.uniprot.org/, accessed on 24 December 2024).

### 2.2. Retrieve Overlapping Targets and Perform Bioinformatics Analysis

The targets of propolis were compared with those related to UV allergic dermatitis to screen out the overlapping targets according to the method in reference [37]. The relationship between these overlapping targets was found through the STRING database (http://string-db.org, accessed on 24 December 2024), and GO and KEGG pathway enrichment analyses were performed using the R (version 4.3.3, R Core Team. 2024. R: A language and environment for statistical computing [Software]. R Foundation for Statistical Computing. https://www.R-project.org/) clusterProfiler package (http://bioconductor.org/packages/release/bioc/html/clusterProfiler.html, downloaded on 22 March 2024). Then, the data exported from STRING database were loaded into Cytoscape software (version 3.9.1; JAVA: 11.0.6 by AdoptOpenJDK) to construct the protein–protein interaction (PPI) network diagram (confidence score ≥ 0.4).

### 2.3. Experimental Verification

#### 2.3.1. Extraction and Preparation of Propolis Sample

The raw-material propolis extraction method and component determination results were the same as in our previous report [38]. Ethanol extract of propolis (EEP, 0.1 g) was dissolved in 0.5 mL of dimethyl sulfoxide (DMSO, Sinopharm Chemical Reagent Co., Ltd., Shanghai, China) and 9.5 mL of DMEM-H cell culture medium (Prosa Life Science and Technology Co., Ltd., Wuhan, China). The propolis solution was stored at −20 °C for further experiments. The aliquoted propolis solution was diluted in half with a cell culture medium to the required concentration.

#### 2.3.2. Model of HSF Cells Treated with UVB Light

Human skin fibroblast-like cells (HSF cells, Prosa Life Science and Technology Co., Ltd., Wuhan, China) were cultured with DMEM-H cell culture medium, 10% fetal bovine serum (Cellmax Bio Co., Ltd., Lanzhou, China), and 1% penicillin and streptomycin mixture (HyClone Biochemical Products Co., Ltd., Shanghai, China) at 37 °C in a humidified cell incubator (C150, Binder, Tuttlingen, Germany) with 5% CO_2_ according to Siyan et al. [39]. The HSF cells at the logarithmic growth phase were digested with trypsin (HyClone Biochemical Products Co., Ltd., Shanghai, China).

A solar dermatitis model was established through UVB irradiation [40]. HSF cells (2 × 10^5^ cells) were added into wells of a six-well plate. The culture medium was removed after the cells adhered to the wall. After washing 2 to 3 times with phosphate-buffered solution (PBS, pH 7.2–7.4, Sinopharm Chemical Reagent Co., Ltd. Shanghai, China), these cells were exposed to FYD-H8A UVB light (UVB-313nm/T12 20W, Fradent (Dalian, China) Medical Technology Co., Ltd., Dalian, China) below 15 cm for 1 min. After irradiation, the cells were washed 3 times with PBS. Then, 10, 20, and 30 μg/mL of propolis solutions (final concentration) and complete culture medium containing 0.015% DMSO (equal to DMSO in 30 µg/mL EEP solution) (blank control) were added in each group with 6 replicates. Cells were cultured for 24 h.

#### 2.3.3. Effect of Propolis on TNF-α, NF-κB, MMP-9, and IL-2 Contents in Culture Medium of UVB-Irradiated HSF Cells

The cell culture medium was centrifuged at 1000× *g* for 20 min. An ELISA assay (reagent kit purchased from Shanghai Enzyme-Linked Biotechnology Co., Ltd., Shanghai, China) was employed to determine the contents of TNF-α, NF-κB, MMP-9, and IL-2 in culture medium [41]. The different concentrations of standard samples (50 μL) were added to the wells in a 96-well plate, the standard group. At the same time, 50 μL of the different concentrations of propolis solutions was added to the sample group. Nothing was added to the blank group. There were 6 replicates for each treatment. Horseradish peroxidase (HRP)-labeled detection antibody (100 μL) was added to the standard, the sample, and the blank control groups. The reaction wells were sealed with a sealing film and incubated in a water bath at 37 °C for 60 min. The liquid was discarded and patted dry with absorbent paper. Each well was filled with washing solution (350 μL) and allowed to stand for 1 min. The washing solution was shaken off and patted dry with absorbent paper. The plate was washed 5 times. Substrates A and B (each for 50 μL) were added to each well and incubated at 37 °C in the dark for 15 min. The stop solution (50 μL) was added to each well. and the OD value of each well was measured at a wavelength of 450 nm using a microplate reader (1510, Thermo Fisher Waltham, MA, USA) within 15 min. The contents of TNF-α, NF-κB, MMP-9, and IL-2 of the samples were calculated based on their OD values and the standard curve obtained with the OD values of the standard group.

#### 2.3.4. The Effect of Propolis on the Proliferation of UVB-Irradiated HSF Cells

Initial CCK-8 assays revealed that propolis maintained cell viability at concentrations below 30 μg/mL, demonstrating favorable biocompatibility without significant cytotoxicity. Therefore, a concentration gradient spanning 10, 20, and 30 μg/mL was established to precisely identify the optimal therapeutic concentration of propolis through dose–response analysis. HSF cells (5 × 10^3^ cells) added 10, 20, and 30 μg/mL of propolis (diluted with complete culture medium) and a blank control (complete culture medium) with 6 replicates in each group were cultured in a cell culture incubator for 24 h. After 24 h, these cells were rinsed with PBS, and then 110 μL of complete culture medium containing 10 μL of CCK-8 solution was added in a dark place. These cells were cultured in a cell culture incubator for 2 h. The absorbance of cells was measured using an enzyme reader at a wavelength of 450 nm. The calculation formula for the proliferation inhibition effect is the following: Proliferation inhibition rate (%) = [(Ac − As)/(Ac − Ab)] × 100%, where As: experimental well (propolis solution of different concentrations); Ac: control well (with cells, culture medium containing CCK-8); and Ab: blank well (no cells, culture medium containing only CCK-8).

### 2.4. Data Analysis

The differences among the TNF-α, NF-kB, MMP-9, and IL-2 contents were analyzed by ANOVA using GraphPad Prism 9.5.1 for Windows (GraphPad Software, Inc., San Diego, CA, USA), and data are presented as (mean ± SE).

## 3. Results

### 3.1. Overlapping Targets of Propolis Components and UV Allergic Dermatitis

**Figure 1:**
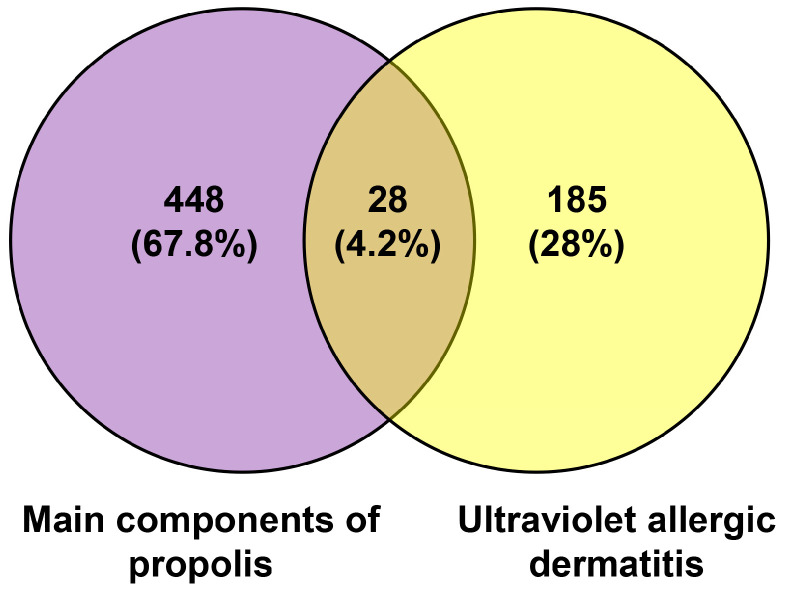
Overlapping targets between the main components of propolis and UV allergic dermatitis.

A total of 448 targets of the main components of propolis (total phenols, total flavonoids) were collected through SEA SearchEMBL-EBI. For UV allergic dermatitis, there were 185 targets. A Venn diagram was drawn for the 185 targets of UV allergic dermatitis and 448 targets of chemical components of propolis (Figure 1). There were 28 overlapping targets for propolis and UV allergic dermatitis: TNF, NFKB1, MMP9, IL2, EGFR, BCL2, EGFR, CASP3, PTGS2, MPO, TLR2, IL5, NOS2, DPP4, BRAF, ALOX5, LGALS3, NLRP3, MALT1, ATM, PDE4A, ADRB2, CFTR, ITGA4, MC1R, CXCR1, CYP27B1, and CCR4.

### 3.2. GO Function Enrichment, KEGG Signaling Pathway Enrichment, and PPI Analysis Results

**Figure 2:**
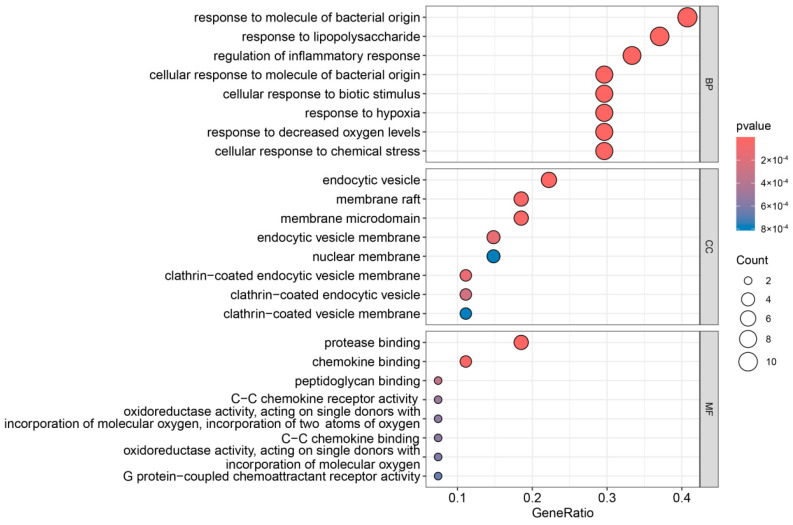
GO enrichment analysis of overlapping targets of the propolis main components and UV allergic dermatitis.

As shown in Figure 2, a total of 1396 relevant items were screened through GO enrichment analysis, of which 1246 items related to biological processes, mainly including response to molecule of bacterial origin, response to lipopolysaccharide, the regulation of inflammatory response, cellular response to molecule of bacterial origin, cellular response to biotic stimulus, cellular response to molecule of bacterial or origin, cellular response to biotic stimulus, response to hypoxia, response to decreased oxygen levels, and cellular response to chemical stress. There were 52 items related to cellular components, mainly including the endocytic vesicle, membrane raft, membrane microdomain, endocytic vesicle membrane, nuclear membrane, clathrin-coated endocytic vesicle membrane, clathrin-coated endocytic vesicle, and clathrin-coated vesicle membrane. There were 98 items related to molecular functions, such as protease binding, chemokine binding, peptidoglycan binding, C-C chemokine receptor activity, oxidoreductase activity, acting on single donors with the incorporation of molecular oxygen, the incorporation of two atoms of oxygen, C-C chemokine binding oxidoreductase activity, acting on single donors with the incorporation of molecular oxygen, and G protein-coupled chemoattractant receptor activity.

**Figure 3:**
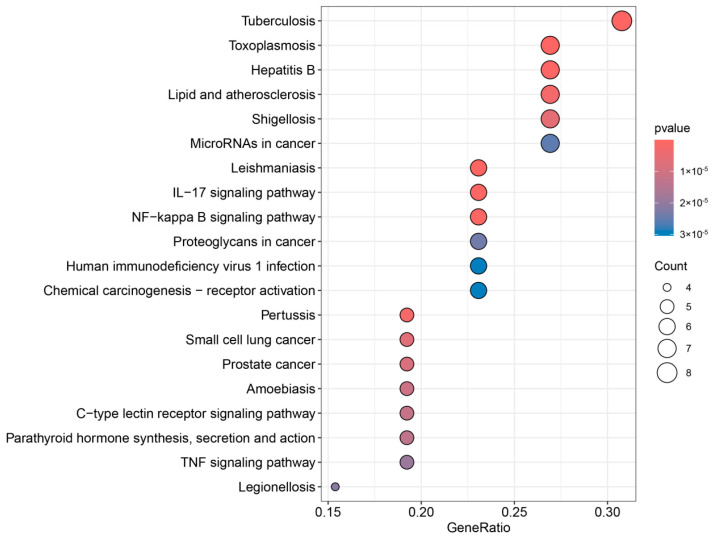
KEGG enrichment analysis of overlapping targets of propolis main components and UV allergic dermatitis.

The 110 common targets related to the main active ingredients of propolis and UV allergic dermatitis were enriched in the KEGG pathway (p adjust < 0.05). The top 20 pathways were screened out (Figure 3), which were closely related to Tuberculosis, Toxoplasmosis, Hepatitis B, lipids and atherosclerosis, Shigellosis, MicroRNAs in cancer, Leishmaniasis, the IL-17 signaling pathway, the NF-kappa B signaling pathway, Proteoglycans in cancer, human immunodeficiency virus 1 infection, chemical carcinogenesis receptor activation, Pertussis, small-cell lung cancer, prostate cancer, Amoebiasis, the C-type lectin receptor signaling pathway, parathyroid hormone synthesis, secretion and action, the TNF signaling pathway, and Legionellosis.

**Figure 4:**
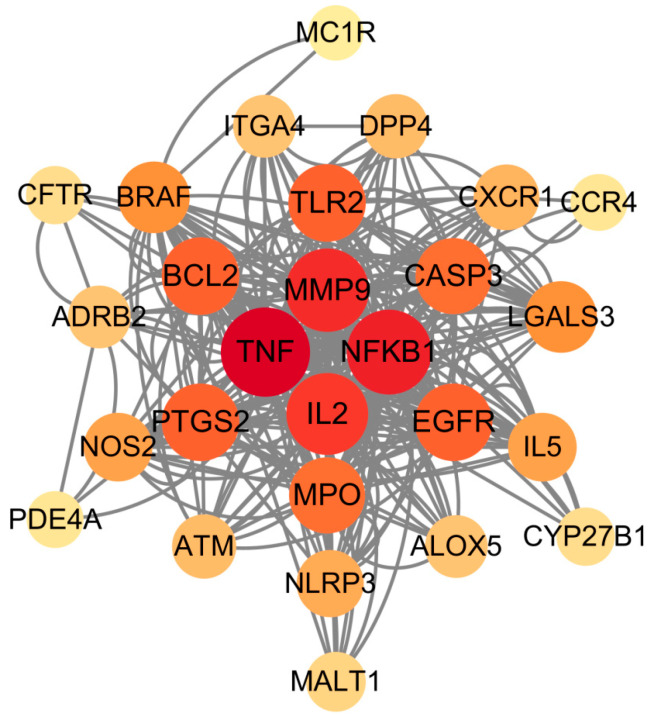
PPI analysis of common targets of the propolis main components and UV allergic dermatitis. The characters indicate the gene names of differentially expressed proteins, and the lines between genes indicate that the differentially expressed proteins interact. The darker the red color and the larger the diameter, the more associated proteins it has.

Among the overlapping targets between the main components of propolis and UV allergic dermatitis, the protein–protein interaction with a confidence level of 0.5 is shown in Figure 4, of which hub proteins were TNF, NFKB1, MMP9, and IL2.

### 3.3. The TNF-α, NF-κB, MMP-9, and IL-2 Contents in Culture Medium of UVB-Irradiated HSF Cells

**Figure 5:**
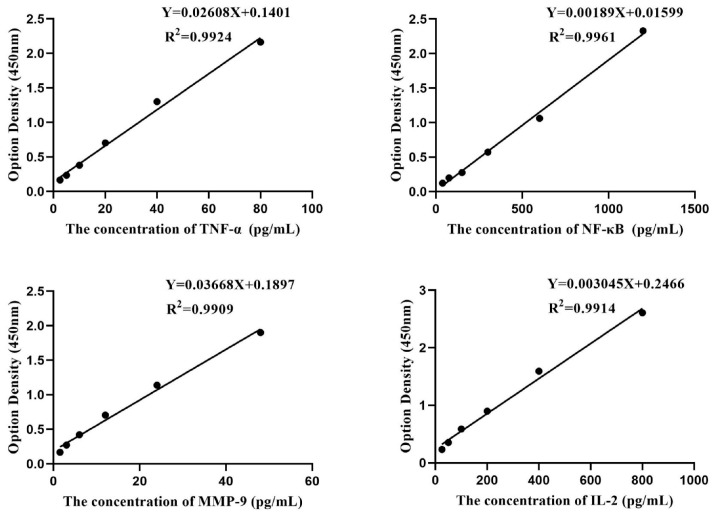
The standard curves of TNF-α, NF-κB, MMP-9, and IL-2 determined by ELISA assay.

**Figure 6:**
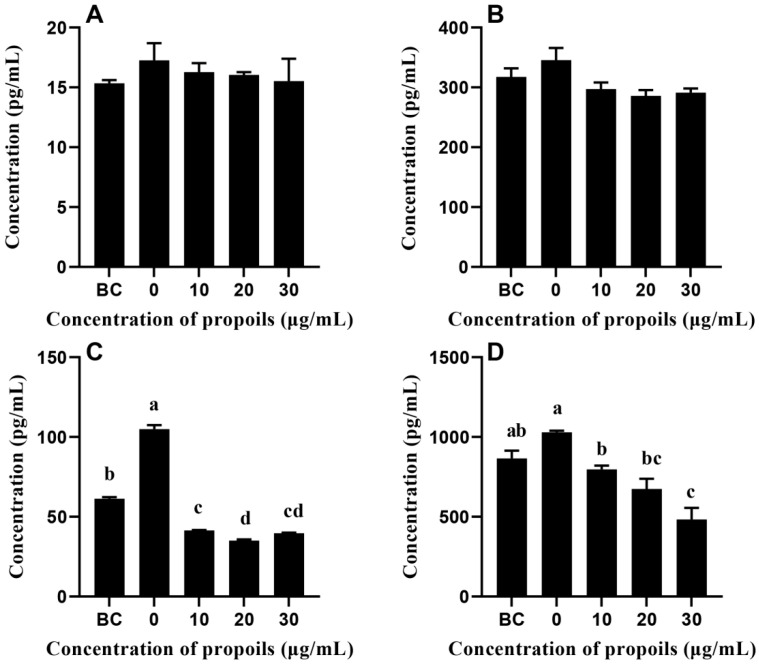
The contents of hub proteins in UVB-irradiated HSF cells: (**A**) TNF-α, (**B**) NF-κB, (**C**) MMP-9, and (**D**) IL-2. Different characters in the figures indicate significant differences between the treatment group and the blank control (BC) group.

The standard curves of TNF-α, NF-κB, MMP-9, and IL-2 are shown in Figure 5. As shown in Figure 6, the contents of MMP-9 and IL-2 in UVB-induced HSF cells were significantly higher than those in untreated cells. Compared with the UVB-induced group, the propolis group significantly reduced the levels of MMP-9 and IL-2 in the cells.

### 3.4. Propolis Affects the Proliferation of UVB-Irradiated HSF Cells

**Figure 7:**
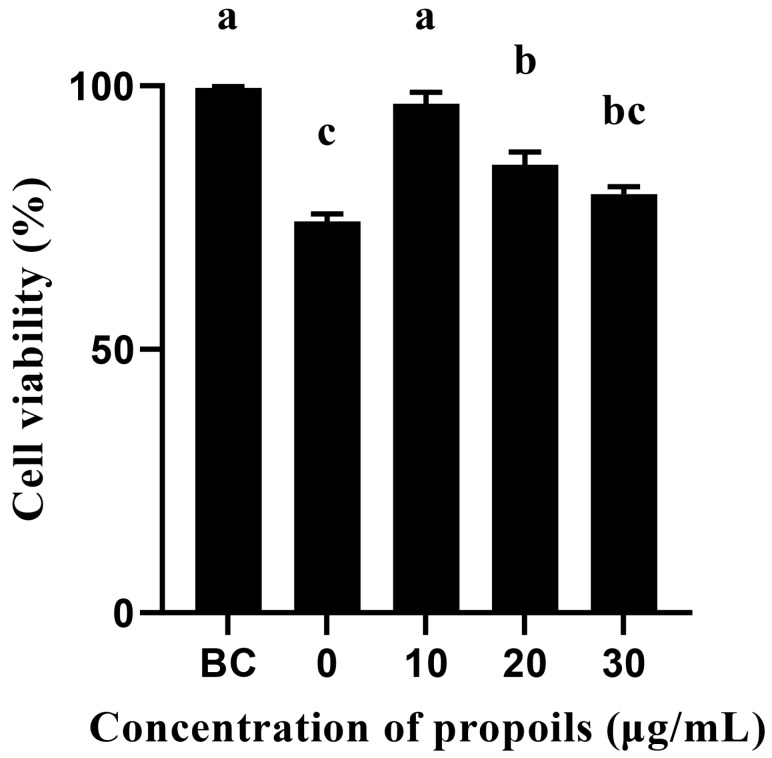
The proliferation of UVB-irradiated HSF cells. Different characters in the figures indicate significant differences between the treatment group and the untreated blank control (BC) group.

As shown in Figure 7, the survival rate of UVB-irradiated HSF cells without propolis was 74.28% of unirradiated blank control group cells. When the propolis addition amounts were 10, 20, and 30 μg/mL, the cell survival rates were 96.65%, 85.07%, and 79.48%, respectively.

## 4. Discussion

As a broad prospect and low-cost research method that combines systems biology and multi-omics analysis, network pharmacology can comprehensively analyze the mechanism of action of complex drug systems by constructing a “component–target–pathway” network [36]. Network pharmacology was employed to predict the function and the targets, which were validated in further experiments [42,43]. As a natural product, propolis has multiple biological activities, such as anti-inflammatory, antioxidant, and immunomodulatory effects. In this study, network pharmacology was employed to investigate the potential mechanism of propolis in treating UV allergic dermatitis.

Some of the overlapping targets between propolis and UV allergic dermatitis were enriched in the TNF signaling pathway, C-type lectin receptor signaling pathway, and parathyroid hormone synthesis, secretion, and action pathways. The TNF signaling pathway is an important pathway for skin inflammatory response and is closely related to the release of inflammatory factors induced by 2,4-dinitrochlorobenzene [44]. Propolis can effectively inhibit TNF-α-induced interleukin (IL)-6 and IL-8 secretion in HaCaT keratinocytes [45]. In addition, the C-type lectin receptor signaling pathway is related to immune regulation. C-type lectin receptor deficiency induces hypersensitivity to infection. Propolis may reduce the sensitivity of the skin by regulating the innate immune response [46,47]. The parathyroid hormone pathway, which is a control of the keratinocyte differentiation and proliferation and angiogenesis of the skin [48], provides indirect support for the treatment of propolis on ultraviolet allergic dermatitis. However, the specific mechanism of action of these pathways still needs to be further verified through in vivo and in vitro experiments.

Some core targets are mainly enriched in biological processes such as the response to bacteria-derived molecules, the response to lipopolysaccharide, the regulation of inflammatory response, the response of cells to bacteria-derived molecules, the response of cells to biological stimuli, the response to hypoxia, the response to reduced oxygen content, and the response of cells to chemical stress pathways. Among them, lipopolysaccharide (LPS) is a typical inflammatory-inducing factor that synthesizes and releases a variety of cytokines and inflammatory mediators through the cell signal transduction system, thereby causing a series of responses in the body [49]. Chrysin in propolis was used to treat LPS-induced septic rats and can reduce the levels of oxidative stress markers and cytokines in sepsis patients [50]. Propolis may inhibit the excessive release of inflammatory mediators and reduce the level of inflammation in UV allergic dermatitis by regulating the response to lipopolysaccharide. The regulation of inflammatory response is a key process in the treatment of UV allergic dermatitis. The main components of propolis may inhibit the expression of proinflammatory factors such as TNF-α and IL-6 [28]. In addition, propolis regulates the response of cells to biological stimuli and bacteria-derived molecules, which may further improve the skin’s tolerance to external stimuli by enhancing the skin barrier function or regulating the local immune microenvironment [51]. Propolis may promote the survival and functional repair of skin cells under hypoxic conditions by regulating the hypoxia-inducible factors (HIFs)-related pathway, which was also found in the weak ultraviolet Ray-B-induced barrier dysfunction of HaCaT cells [52]. This regulatory mechanism is of great significance for tissue repair and functional recovery in ultraviolet allergic dermatitis. In addition, propolis may remove ultraviolet-induced reactive oxygen species (ROS) through antioxidant mechanisms, thereby reducing oxidative stress damage to the skin, which was also found in the UVA-induced apoptosis of human keratinocyte HaCaT cells [53]. Propolis can reduce the inflammatory response in HSF cells induced by UV allergic dermatitis by modulating multiple signal pathways.

The results of PPI analysis showed that TNF (tumor necrosis factor) with the highest significance is a key mediator of inflammatory response. It can promote the release of inflammatory factors by inducing the activation of downstream signaling molecules (such as NF-KB1), which is an important transcription factor that regulates immune and inflammatory responses, thereby aggravating inflammatory damage to the skin [54]. The abnormal activation of NF-KB1 is often associated with inflammatory skin diseases [55]. MMP-9 is a matrix metalloproteinase that can degrade the extracellular matrix and affect the integrity and repair ability of the skin barrier [56]. IL-2 (interleukin 2) also plays a key role in regulating the activity of immune cells. Its overexpression may aggravate local inflammatory responses [57]. Additionally, there are also other proteins, such as EGFR, BCL2, CASP3, PTGS2, and MPO, that contribute to the anti-inflammatory response. The activation of EGFR (epidermal growth factor receptor) is closely related to the repair and regeneration of the skin. Propolis may play a positive role in promoting skin damage repair by regulating the signaling pathway of EGFR [58]. BCL2 and CASP3 play important roles in the inhibition and induction of apoptosis, respectively, and their dynamic balance is crucial for the survival and renewal of skin cells [59,60]. Studies have found that propolis may inhibit excessive apoptosis by regulating these key targets, promoting skin repair [61]. In addition, PTGS2 (cyclooxygenase 2) is an important enzyme in the inflammatory pathway. Its overexpression increases the production of proinflammatory prostaglandins, thereby exacerbating the inflammatory response. Propolis may relieve inflammation by inhibiting PTGS2 activity [62]. MPO (myeloperoxidase) is an important marker of neutrophil activation, which plays a role in oxidative stress and inflammatory response [63]. The upregulation of MMP-9 and IL-2 after UVB induction may be closely related to skin inflammation and immune abnormalities [64]. After propolis treatment, the levels of MMP-9 and IL-2 were significantly reduced, indicating that propolis may reduce UV-induced skin damage by regulating matrix degradation and immune inflammatory response. This is consistent with the known anti-inflammatory and immunomodulatory effects of propolis [28]. Related experiments have shown that propolis may inhibit the expression of TNF-α and MMP-9 and relieve inflammation by regulating NFKB1 signaling, and propolis can directly promote the transcription of IL-2 and MMP-9 by regulating NFKB1 signaling [65,66].

The results of the HSF cell survival experiment showed that UVB irradiation significantly reduced the survival rate of HSFs cells. However, when different concentrations of propolis (10, 20, 30 μg/mL) were added, the cell survival rates increased to 96.65%, 85.07%, and 79.48%, respectively, indicating that propolis has a protective effect on UVB-induced cell damage. Among them, the growth effect was most significant when the propolis concentration was 10 μg/mL, making the cell survival rate close to the normal level. As the propolis concentration increased, the survival rate showed a downward trend, which may indicate that high concentrations of propolis have a certain cytotoxic or inhibitory effect on HSF cells. This phenomenon is consistent with existing studies, that is, the biological effects of propolis are usually dose-dependent, but at higher concentrations, excessive components or metabolic stress may lead to a decrease in cell adaptability or the generation of toxic effects. Brazilian propolis not only reduced periodontal ligament (PDL) cell apoptosis but also increased the metabolic activity and proliferation of PDL cells [67]. The pyruvate/chitosan/propolis fiber nanocomposite scaffold has good biocompatibility for HSF cells [68]. As the propolis concentration increased further, the cell proliferation rate was not as high as that at low propolis concentrations. The results showed that there was no statistical difference between Brazilian green propolis and its control solution when the propolis concentration was 0.12 to 7.81 μg/mL; however, at a concentration of 31.25 μg/mL or higher, propolis was toxic to NIH-3T3 cells [69].

In summary, propolis has demonstrated promising potential in alleviating inflammation, promoting the tissue repair and functional recovery of skin cells, and enhancing the proliferation of HSF cells in UV allergic dermatitis, despite the current lock of in vivo experiments validation.

## 5. Conclusions

This study systematically explored the potential mechanism of propolis in treating UVB-induced allergic dermatitis using network pharmacology and in vitro experiments. The results revealed the significant effects of propolis in biological processes (1246 items), cellular components (52 items), and molecular functions (98 items) through GO functional enrichment analysis. In addition, KEGG signaling pathway enrichment analysis showed that propolis was significantly associated with 110 signaling pathways. There were 28 core targets related to UVB-induced allergic dermatitis, of which hub proteins were TNF-α, NFKB1, MMP-9, and IL-2. Elisa’s experimental verification found that propolis could significantly reduce the levels of inflammatory factors MMP-9 and IL-2 in a dose-dependent manner. Propolis promoted the proliferation of UVB-irradiated cells. This research provided the pharmacological functions of propolis and the promotion and application of natural products as a functional food, dietary supplements, or medicinal agent in the treatment of UVB-induced allergic dermatitis.

## Data Availability

The original contributions presented in the study are included in the article, further inquiries can be directed to the corresponding author.
